# The promoting effects of activated olfactory ensheathing cells on angiogenesis after spinal cord injury through the PI3K/Akt pathway

**DOI:** 10.1186/s13578-022-00765-y

**Published:** 2022-03-04

**Authors:** Xiaohui Wang, Chao Jiang, Yongyuan Zhang, Zhe Chen, Hong Fan, Yuyang Zhang, Zhiyuan Wang, Fang Tian, Jing Li, Hao Yang, Dingjun Hao

**Affiliations:** 1 Department of Spine Surgery, Hong Hui Hospital, Xi’an Jiaotong University, Xi’an, 710054 China; 2 Department of Developmental Genetics, Max Planck Institute for Heart and Lung Research, 61231 Bad Nauheim, Germany; 3 Department of Neurology, The Second Affiliated Hospital of Xi’an Jiaotong University, Xi’an, 710004 China; 4 Department of Medicine, Solna, Karolinska Institutet, 171 77 Stockholm, Sweden; 5 Department of Orthopaedic, The Second Affiliated Hospital of Xi’an Jiaotong University, Xi’an, 710004 China; 6 Translational Medicine Center, Hong Hui Hospital, Xi’an Jiaotong University, Xi’an, 710054 China

**Keywords:** Olfactory ensheathing cells, Spinal cord injury, Angiogenesis, Cell transplantation, Endothelial cells, PI3K/Akt

## Abstract

**Objective:**

The aim of this study was to investigate the pro-angiogenic potential of olfactory ensheathing cells (OECs) activated by curcumin (CCM) and lipopolysaccharide (LPS) and the possible underlying mechanisms.

**Methods:**

Vascular endothelial cells or tissues were cultured and treated with conditioned medium (CM) extracted from activated OECs activated through the addition of LPS and CCM or unactivated controls. Concomitantly, the pro-angiogenic potential of OECs was assessed in vitro by aortic ring sprouting assay, endothelial wound healing assay, CCK-8 assay, and tube formation assay. Subsequently, the OECs were co-cultured with endothelial cells to evaluate their promoting effect on endothelial cell proliferation and migration following a mechanical scratch. Moreover, the spinal cord injury (SCI) model in rats was established, and the number of endothelial cells and vascular structure in the injured area after SCI was observed with OEC transplantation. Finally, the underlying mechanism was investigated by western blot analysis of phosphorylated kinase expression with or without the MK-2206 (Akt-inhibitor).

**Result:**

The present results showed that the activated OECs can effectively promote vascular endothelial cells' proliferation, migration, and vessel-like structure formation. Strikingly, several pro-angiogenic growth factors such as VEGF-A and PDGF-AA, which facilitate vessel formation, were found to be significantly elevated in CM. In addition, the PI3K/Akt signaling pathway was found to be involved in pro-angiogenic events caused by activated OEC CM, displaying higher phosphorylation levels in cells. In contrast, the delivery of MK2206 can effectively abrogate all the positive effects.

**Conclusions:**

OECs activated by LPS and CCM have a pro-angiogenic effect and can effectively promote angiogenesis and improve the microenvironment at the injury site when transplanted in the injured spinal cord. This potentiated ability of OECs to provide pro-angiogenic effects is likely mediated through the PI3K/Akt pathway.

**Supplementary Information:**

The online version contains supplementary material available at 10.1186/s13578-022-00765-y.

## Introduction

Spinal cord injury (SCI) can cause a permanent loss or reduction in motor and sensory functions below the damaged segment, subsequently leading to axonal and cellular damage, along with ischemic changes and inflammatory infiltrates [1, 2]. Cells are complex systems with multiple targets and stimuli-responsive functions. Therefore, cells from several different tissue sources have great potential to treat patients suffering from SCI. Importantly, cell therapies’ multiple targets and stimuli-responsive functions can improve SCI regeneration by regulating inflammatory responses, providing nutritional support, neuronal replacement, and axon remylein [3]. These characteristics of cell therapies align well with the preceding complex pathological changes of SCI as their therapeutic targets.

Olfactory ensheathing cells (OECs) can induce neuroplasticity/neuroregeneration following SCI, thereby contributing to these cells being intensively studied as potential candidates for cell transplant therapy of SCI [4, 5]. These novel investigations have mainly explored OEC functions and/or aimed to strengthen their targeted functions. Notably, several bioactive factors derived from OECs have been found to indirectly improve the microenvironment of injured areas. OECs have also been found to have direct interactions and crosstalk with astrocytes, influencing glial scar reconstruction and aiding in beneficial phagocytosis [4, 6–10]. Collectively, these OEC functions promote inflammatory regulation and induction of neuronal migration and survival via neurotrophic factors in injured areas, which can potentially provide nerve repair through microenvironmental factors. However, there are several limitations in the application of OEC transplantation that may result from survival rates after transplantation, OEC purity, and the anatomical source [11, 12]. Therefore, to maintain the functional stability and develop the therapeutic potential of OECs, optimizing the sampling methods and source of OECs and improving pretreatment of cultured OECs prior to transplantation will significantly enhance the ability of OECs to regulate the microenvironment in SCI [13, 14]. In relation to these findings, we recently observed that OECs had improved bioactivity with the pretreatment of lipopolysaccharide (LPS) and/or curcumin (CCM), with improvement observed in phagocytic capacity and promotion of neuronal growth [14–16].

The maintenance of blood supply is an essential basis of microenvironmental homeostasis. In contrast, primary mechanical damage resulting from SCI disrupts the topical capillaries and the blood–brain-spinal cord barrier (BSCB). As a consequence of these responses, ischemic changes occur in the spinal cord due to inflammation, edema, and vascular endothelial cells damage [17]. Under the continuing effects of these adverse changes and other secondary injuries, the new functional vessels in and adjacent to the injury epicenter are unable to form sufficiently [18]. Both axon regeneration and inflammatory product removal depend on the vascular system, so there is no doubt that ischemia can exacerbate the injured area’s microenvironment and increase the difficulty of neurological recovery [19, 20]. Therefore, the promotion of blood-supply regeneration should not be neglected in exploring the conditions of nerve recovery after SCI. Additionally, amelioration of angiogenesis should be considered the primary strategy for early intervention, as this function is critical to improving regeneration and inflammatory product clearance and providing a nutritional foundation [21–23].

Although there are many studies on the effects of OEC transplantation on improving microenvironment and nerve recovery after SCI, few investigations have reported the effects of OECs on vascular endothelial cells or the spinal vascular system. From what has been investigated, OECs have been found to express vascular endothelial growth factor (VEGF), which is a growth factor effective in promoting angiogenesis [24–26]. These preliminary studies have revealed the potential of OECs to promote angiogenesis, thereby identifying a promising therapeutic approach that can potentially improve vascular system injuries of the spinal cord.

Previously, investigations elucidating the benefits of OEC transplantation have identified increased vascular structure and directional angiogenesis in animal models of SCI following treatment with OEC transplantation [27, 28] However, due to the complexity of the in vivo environment and their targeted area of research, the authors did not systematically describe any behavioral changes and/or related mechanisms of angiogenic stimulation induced by OECs. Therefore, the function of OECs in angiogenesis remains to be elucidated.

To address these gaps, we systematically studied the changes of vascular endothelial cells and vascular tissues after OEC intervention in vivo and in vitro, involving indirect (conditioned media for OECs) and direct effects (in vitro co-culture and in vivo cell transplantation). In addition, based on the growth factor array screening results in conditioned medium, we identified possible molecular pathways potentially involved in vascular tissue modifications resulting from OEC transplantation. Furthermore, based on conventional isolation and culture of OECs, we also used OECs pretreated with LPS and CCM, previously reported to act as activators, to explore whether the pretreated OECs had improved responses than untreated OECs [15, 16].

## Materials and methods

### Main reagents

Dulbecco’s modified Eagle’s medium (DMEM)/F12, fetal bovine serum (FBS), 0.25% EDTA-trypsin, DMEM, and penicillin/streptomycin solution were purchased from Gibco (Carlsbad, CA, USA); LPS, CCM, poly-Lysine (PLL), dimethyl sulfoxide (DMSO), bovine serum albumin (BSA), 0.2% gelatin, and crystal violet dye were purchased from Sigma-Aldrich (St. Louis, MO, USA); Dispase (neutral protease, grade II) was purchased from Roche (Basel, Switzerland); Anti-p75 antibody, anti-CD31 antibody, anti-pan-Akt antibody, anti- PI3K p85 α antibody, anti-phospho-PI3 K p85α (Y607) antibody and 5-ethynyl-2-deoxyuridine (EdU) Proliferation Kit (iFluor 488) were purchased from Abcam (Cambridge, MA, USA); Anti-phospho-Akt (Ser473) antibodies were purchased from Cell Signaling Technology (CST; Danvers, MA, USA). Human umbilical vein endothelial cells (HUVECs), endothelial cell medium (ECM), and endothelial cell growth supplement (ECGS) were purchased from Sciencell Research Laboratories (Carlsbad, CA, USA); Red Fluorescent Probe M02 kit was purchased from Bestbio (Shanghai, China); MK-2206 was purchased from Selleck Chemicals (Houston, TX, USA); Cell counting kit-8 (CCK-8) proliferation assay, western blotting kit, and anti-β-actin antibody were purchased from Boster Biological Technology (Wuhan, Hubei, China); DAPI Kit, Fluor594-conjugated goat anti-rabbit IgG and Fluor594-conjugated goat anti-mouse were purchased from Molecular Probes (Eugene, OR, USA). Anti-mouse/rabbit immunohistochemistry detection kit was from Proteintech Group (Cambridge, MA, USA). MICROFIL® angiography kit was purchased from Flow Tech Inc. (Carver, MA, USA). 35 mm dishes, cell culture plates, plastic coverslips, and flasks were all purchased from Thermo Fisher (Shanghai, China). Matrigel basement membrane matrix and 24-well Transwell plates were purchased from Corning (Corning, NY, USA); 35 mm confocal dishes and centrifuge tubes were from NEST (Wuxi, Jiangsu, China). Microcentrifuge tubes (EP tubes) were purchased from Eppendorf (Hamburg, Germany).

### Primary culture, purification, and identification of OECs

All experimental protocols involving experimental animals used in this study were reviewed and approved by the ethics committee at Honghui Hospital, affiliated with Xi’an Jiaotong University, and conformed to the Guide for the Care and Use of Laboratory Animals published by the National Institutes of Health. As previously described, OECs were isolated and primarily cultured from olfactory blubs in rats at 2–3 months of age (Additional file 1: Fig. S1) and further purified by the modified differential adherent velocity method as published by H. Nash et al. [15, 29]. Minor modifications to this protocol included efficient removal of the tractus olfactorius (inner layer), meanwhile maintaining the outer olfactory bulb layer (nerve layer and glomerulus layer) based on the distribution characteristics of OECs, and to eliminate interference from other glial cells [13]. After purification, the OECs were reseeded onto 25-cm^2^ PLL-coated culture flasks and maintained in DMEM/F12 media with 10% FBS, cultured at 37 °C and 5% CO_2_ in an incubator (Thermo Fisher, Waltham, MA, US). Half of the total volume replaced every 3 days. Typically, OECs would reach about 80% confluence 10 days after isolation and seeding. OECs were then identified by immunofluorescence staining of anti-p75 antibody or activated by CCM & LPS [14]. Enhanced green fluorescent protein (GFP) transgenic rats were selected as the olfactory bulb donor when we prepared OECs for in vitro co-culture and in vivo transplantation. This modification to the previous protocol allowed us to observe the distribution of OECs more intuitively [14].

### Activation of OECs and conditional-media (CM) collection

When the OECs reached optimal confluence, cells were digested by 0.25% EDTA-trypsin and made into suspensions, divided equally into two groups, and then reseeded on 6-well plates (for experimental operation) or coverslips (for immunofluorescence identification). A same batch of OECs is usually divided equally, and one designated group of OECs is activated randomly as the activated group.One group was noted as Activated-OECs (Act-OECs), the media for this group contained 1 μg/ml LPS and 1 μM CCM dissolved in DMSO as previously described by Hao et al. [14]. The other group was noted as Unactivated-OECs (Una-OECs), in which this group’s media contained equal amounts of DMSO as a control (the solvent used for CCM and LPS). To investigate whether the OECs could promote angiogenesis of vascular endothelial cells, we collected the conditioned media of OECs from cell culture, in which DMEM/F12 media with 10%FBS was used as the no-treatment control group. After one day of treatment, the media mixed with drugs or DMSO were removed and replaced with complete media. To ensure adequate nutrients, conditioned media were collected once every other day after the day of media replacement, a total of three times. Three days later, OEC supernatants were combined, mixed, and centrifuged at 14,000 × g for 15 min at 4 °C and then filtered with sterile 0.22-μm pore membranes to remove cell debris. Finally, these collected conditioned media isolates were kept at − 80 °C until used.

### Rat aortic ring assay (Neovascularization)

This operation is performed based on previously published protocols [30, 31]. Rat aortic rings were isolated from young rats (three-weeks-old, weighing 50 g ± 10 g) and incubated in serum-free DMEM overnight as starved pretreatment. Before the aortic ring sprouting assay, 100 μL of Matrigel was added to each well of a 96-well plate by precooled pipette tip, with one aortic ring per well embedded in the middle of the gel. Following this step, plates were incubated in a 37 ℃ cell incubator for half an hour to allow for the gel to set. (Additional file 2: Fig. S2b) Once the gel was set, 150 µL ACM, UCM, or control media with 10%FBS was added to each well according to their respective groups, in which each group had three replicates. These aortic rings were cultured at 37℃ and 5% CO_2_ for 7 days, and then the sprouting results were photographed under an inverted phase-contrast microscope (Leica DM IL; Leica Microsystems, Germany). Each test was repeated three times, and relevant sprouting indexes were digitally calculated using Image J software (National Institutes of Health, Rockville, MD, USA).

### Endothelial wound healing (cell migration) assay

Migration of endothelial cells was determined using a two-dimensional wound-scratch assay on 24-well plates. HUVECs were seeded onto each well of 24-well plates (1 × 10^5^ cells/well) and maintained in DMEM with 10% FBS for 24 h, followed by overnight starvation with serum-free media. At the bottom of each hole, adherent cells were scratched radially through the center by 10 μL pipette tips, and then each hole was washed with phosphate-buffered saline (PBS) three times to remove the floating cells, allowing for the wound to be immediately seen under a microscope. Next, the HUVECs were cultured in 500 μL ACM or UCM or control media, respectively. To examine the motility contributing to the healing and exclude components related to cell proliferation, HUVECs were incubated with the antimitotic agent mitomycin (Sigma-Aldrich, USA). The wounds were observed using an inverted phase-contrast microscope after 24 h. Photographs were taken at regular intervals over 24 h until wound closure was achieved. The operation was repeated three times, and the results were analyzed with Image J. Healing index = (initial area—final area)/ initial area × 100%.

### CCK-8 proliferation assay

The cultured HUVECs were digested and centrifuged, and the liquid supernatant was removed. After collecting the supernatant, 100 μL was dispersed uniformly with respect to the experimental groups, and the HUVEC suspension was distributed and inoculated to each well (5000 cells/well) in a 96-well plate. The HUVEC distributed plates were incubated for 1 h (37 °C, 5% CO_2_) until cells were adherent. Following adhesion, 10 μL of CCK-8 solution was added to each well of the plate and then incubated for 6 h (37 °C, 5% CO_2_). The plates were measured for absorbance every hour at 450 nm throughout the incubation period using a microplate reader (Thermo Fisher, Waltham, MA, US), and the OD_450_ values were recorded.

### HUVECs tube formation assay

Matrigel was coated in each well of a precooled 96-well plate (50 μL/well) and incubated at 37 °C for 0.5 h. Following Matrigel coating, 3 × 10^4^ HUVEC cells/well were seeded within the designated media of preset groups on Matrigel as illustrated in Additional file 2: Fig. S2C and previously described. The tube formation ability of HUVECs was measured at 3, 6, 9 h. After incubation, images were captured by an inverted phase-contrast microscope. The capillary length and the number of nodes/junctions/meshes of the tubular structures were quantified by Image J, and these processes were independently repeated as triplicates.

### Isolating and primary culture of rat aortic endothelial cells (RAECs)

The aorta of young rats (three-week-olds, weighing 50 g ± 10 g) were isolated and obtained under aseptic conditions. Under an anatomical microscope (OPTON, German), the clot and connective tissue are stripped from the aorta using ophthalmic microscopic surgical instruments. After being washed by DMEM with 1% penicillin/streptomycin three times, a surgical suture was threaded through the active vessels, and the ends of the vessels were ligated. (Additional file 3: Fig. S3A–E). The intima of the aortas were turned over and exposed by suture-pulling. The unligated end was closed by a vascular clamp. (Additional file 3: Fig. S3F) The aorta was then incubated in 1 mg/ml Dispase for 60 min to digest the collagen in the cell–matrix in this inverted state of the intima. Next, the aorta was transferred to DMEM with 10% FBS and dissected into small 2 mm × 2 mm slices. These slices were placed at the bottom of the gelatin-coated 6-well plate (ensuring that the intima faces downward) with 1–2 samples per well, as shown in Additional file 3: Fig. S3G. During the time that the tissues were not adherent to the plate, the medium was maintained to moisten the bottom of the samples without flooding it. The amount of ECM with 5% FBS and 1% EGS added at each well during this process was about 800 μL. Following these steps, the 6-well plates were placed in a small wet box and cultured at 37 ℃, 5% CO_2_, and the media was changed every 48 h. 4–5 days after incubation, RAECs can be seen extending out from the tissue margins under a microscope. The tissue slices were removed at this stage, and 2 mL of media was added to each well, with media being replaced every other day. After day 10, the RAECs could be subcultured, cryopreserved, or used for further experiments. To validate these cell types, cultured cells were identified by immunofluorescence using an anti-CD31 antibody.

### Co-culture of OECs and RAECs in vitro

After primary cell culturing, collections, and mixing, the RAECs were reseeded in each gelatin-coated well of a 24-well plate (1 × 10^5^ cells/well) using DMEM/F12 media with 10% FBS for 2 h to allow the cells to adapt to the new environment. The original medium was then replaced with fresh media containing 0.5% red fluorescent probe M02. Two hours after medium replacement, the cells were labeled with fluorescence, washed with PBS three times, and replaced with fresh medium without a probe. Similar to the wound healing—cells migration assay, the wounds were generated by manually scratching the cell surface with 10 μL pipette tips, which can mimic the conditions of vascular endothelial injury in vivo. After that, suspension of pre-digested activated OECs or unactivated OECs were added into thewells of different groups, respectively. Wells containing medium without cells was set as a no-treatment control. All steps were repeated in triplicates. Similar to previous methods [32], before and after manual scratch, co-culture for 1 h and co-culture for 24 h was set as the shooting time, and the wound healing was analyzed using merged images of red fluorescence (M02: RAECs), green fluorescence (GFP: OECs) and bright-field (all cells) imaging. The healing index was calculated as described above.

### Animal and experimental setting

Forty-eight female Sprague–Dawley rats weighing 200 ± 30 g (specific pathogen-free) were obtained from the Laboratory Animal Centre of Xi’an Jiaotong University. The rats were housed at constant temperature (23 ± 2 °C) and humidity of 50% ± 10% on a 12/12 h light/dark cycle with constant air renewal. The animals were divided into four groups at random as follows: (i) in the sham group, rats were subjected to laminectomy but not SCI; (ii) in the SCI group, rats were subjected to laminectomy and compressional SCI; (iii) in the activated-OECs transplantation group (AOT), a concentrated suspension of activated OECs were transplanted based on the SCI group; (iv) in the unactivated-OECs transplantation group (UOT), a concentrated suspension of unactivated OECs was transplanted based on the SCI group. Each group contained 12 experimental animals, and behavior and morphology were observed after the operations mentioned above.

### Animal SCI model and OECs therapy

Twenty minutes before surgery, rats in each group were anesthetized using isoflurane and a veterinary anesthesia machine (RWD, Shenzhen, Guangdong, China). After isoflurane treatment, the rats were then anesthetized intraperitoneally with 1% sodium phenobarbital (4 mL/kg). Concomitantly, the rats were immobilized in the prone position. Vaseline oil was used on the eyes to prevent drying during surgery. After removing the hair of the animals, the operational locations were disinfected with medical iodine volts. The operational method for the compressional SCI model was performed as previously described [33]. Briefly, for one case, a longitudinal incision was performed overlying the T7–11 area using operating scissors under aseptic conditions. After subperiosteal dissection of paraspinal muscles, a laminectomy was performed to expose the spinal cord from T9 or T10 (Additional file 4: Fig. S4a). In the exposed area of its spinal cord, a compressional lesion was produced using a calibrated compression method with microsurgical forceps (Yunkang, Jiangsu, China) for 20 s followed by removing the forceps carefully [34]. The tip spacing of the forceps was 1 mm during compression. (Additional file 4: Fig. S4a–c).

OEC transplantation was performed with the rats positioned in a stereotaxic instrument (RWD). Either 5 μL of a cell suspension containing 1 × 10^5^ cells or saline control (SCI group) was injected into the core site of the injured spinal cord using a 10 μL siliconized Hamilton syringe with a beveled glass pipette tip (80 × 90 mm inner diameter) (Fig. 4a). Injection speed was uniform and slow (0.2 µ l/min), and the glass pipette was left in position for an additional 5 min to prevent leakage before the withdrawal. The whole-cell transplant process took half an hour [15].

Following these steps, we used sterile normal saline to wash out the incisal opening and sew up each tissue layer. Three days after the operation, the rats were fasted but received an appropriate amount of glucose to their water source for energy. Furthermore, they were given daily intraperitoneal injections of cefuroxime to prevent infection. Urination was artificially assisted by massaging the bladders every day, and feeding quantity was gradually adjusted to their behavior and recovery.

### Basso, beattie, and bresnahan (BBB) locomotor scale

The model’s establishment effect and treatment effect were evaluated using the BBB locomotor scale using the methods described in the published literature [35]. The scores for each group were recorded 1 d, 3 d, 7 d, 14 d, and 21d post-injury. The BBB scores for each group were observed, averaged, and recorded by two trainees who were blinded to the groups. The scoring criteria included joint movement, paw placement, and coordination. Two trainees recorded these animal data in a noise-free, open field arena for 5 min at least. The rats with normal motor function obtained a BBB score of 21points, while the rats losing complete motor function were scored 0 points. Finally, the data was compiled and analyzed using a two-sample t-test and linear mixed-effects model analysis.

### Immunofluorescence and immunohistochemistry

OECs and RAECs on plastic coverslips of all groups were fixed with 4% paraformaldehyde (Sigma) for 30 min, and then treated with 3% BSA in 0.01 M PBS as a nonspecific blocker for 30 min. Coverslips were incubated with primary rabbit monoclonal antibodies against p75 (1:500, for OECs) or mouse monoclonal antibodies against CD31 (1:1000, for RAECs), at 4 °C overnight, washed in PBS three times and incubated with the corresponding fluorescence conjugated secondary antibody (Alexa Fluor® 594 goat anti-rabbit IgG and Alexa Fluor® 594 goat anti-mouse IgG antibody, 1:1000 dilution) for 2 h followed by DAPI nuclear staining (1:1000) at room temperature (RT) for 10 min. Coverslips were then inverted onto the glass slides and sealed by antifading mounting medium (Boster) after rinsing in PBS. All cells were primarily cultured independently for three times.

For animal models 7-day post-surgery, after being anesthetized with 1% sodium phenobarbital (4 mL/kg), their left ventricle was washed in saline and perfusion fixed by 4% paraformaldehyde. Spinal cords were isolated from the vertebral canal and fixed in 4% paraformaldehyde at 4 °C for three days, cryoprotected in 30% sucrose in 0.1 M PBS for 1 week, cut into segments, embedded in optimum cutting temperature compound (SAKURA Tissue-Tek, CA, USA), sectioned at 10 mm thickness, and pasted into PLL coated slides for immunofluorescent and histochemical staining analysis.

Immunofluorescence of histological sections was performed following previously described methods of immunofluorescence staining of cells. An additional step was added in which the sections were incubated with 0.01% triton-X100 (Sigma-Aldrich, USA) for 1 h at RT before BSA incubation.

One week after injury, the immunohistochemistry method identified CD31-positive vascular endothelial cells were quantified at the spinal lesion site. Briefly, at RT, the histological sections were incubated with 3% H_2_O_2_ for 10 min to inactivate endogenous enzymes, incubated in BSA for 1 h, incubated overnight at 4 °C with anti-CD31 antibody, and finally washed three times by PBS. After being combined with goat anti-Mouse/Rabbit Poly- horseradish peroxidase (HRP) secondary antibody, 3,3’ Diaminobenzidine Tetrahydrochloride was added to the tissue to produce a chromogenic reaction. After washing, it was redyed with hematoxylin for 2–3 min, then rinsed with distilled water, followed by gradient dehydration with alcohol (60%,75%,100%) for 5 min each. After removal, it was placed in xylene twice for 5 min, sealed with neutral balsam (Bestbio, China), and observed.

All slides were observed under a Leica DM6 B microscope (Leica Microsystems, Germany). All images were captured using the Leica LAS X software (Leica Microsystems, Diegem, Belgium). Results were measured by Image J software.

### Angiography of spinal cord

Paraformaldehyde perfusion fixation was performed on animals as previously described. After that, the circulatory systems of rats were perfused with the Microfil® MV-122 (Yellow) silicone rubber contrast agents. A sign of successful infusion is yellow-dying of the microvessels in the sclera or liver of the animal. Following infusion, the sacrificed animals were stored at 4 °C to promote Microfil in the blood vessels to fully polymerize. The spinal cords were extracted and cut to remain 1.5 cm (centered around the lesion). (Additional file 5: Fig. S5).

Micro-computed tomography (micro-CT) images were obtained using eXplore Locus SP system (General Electric, Milwaukee, WI, USA), and the resolution was set at 5 microns. The angiographic images were reconstructed to 3D vascular models and analyzed by VGStudio MAX (Volume Graphics, Heidelberg, Germany).

### Growth factor assay

The growth factors in the CM of OECs were semi-quantitatively evaluated using a multiplex growth factor array system (Rat Growth Factor Array 1; RayBiotech, Norcross, GA, USA). CM dilution was not necessary. However, the collected CM was calibrated according to cell numbers. All processes were operated according to the user manual. The chemiluminescence signal of each membrane dot was imaged using a ChemiDoc XRS (Bio-Rad, Hercules, CA, USA) and quantified using Quantity One software (Bio-Rad) which allowed for the assessment of growth factor content in the CM. Tests were repeated as triplicates. Collected CM came from different OEC-culture batches, and results were measured using the Image J software.

### Western blots

Based on the result of the growth factor assays, intervention was performed on three groups that had been set up for in vitro experiments. In each group, MK2206 was dissolved into a DMSO solution and added into media to achieve a final concentration of 5 μM. Three groups received MK2206, and three control groups received equal amounts of DMSO as a control for culturing HUVECs, respectively. HUVECs were mixed with grouping media and inoculated into wells corresponding to the group of 6-well plates (10^5^ cells/ well). (Additional file 6: Fig. S6d) After 24 h, Akt and PI3K changes in phosphorylation were analyzed. Total cellular extracts were prepared as follows: HUVECs were carefully and briefly rinsed with saline buffer and extracted in ice-cold RIPA as described by Yang et al. [36]. Notably, phenylmethanesulfonyl fluoride and phosphatase inhibitor (Beyotime, Jiangsu, China) was added into the PIPA (1:50) to prevent protein degradation and dephosphorylation [37]. The protein concentrations of cell lysates were measured using the BCA protein assay (Qiagen, Germany), using BSA as a reference. The following antibodies were used: pan-Akt (1:500), phospho-Akt (1:2000), PI3K p85 α (1:1000), and Phospho-PI3K p85α (1:500). All protein lysates were run on 10% gradient SDS-PAGE for 1.5 h, transferred onto polyvinylidene fluoride (PVDF) membranes for 50 min, blocked with 5% fat-free milk for 1 h at RT for 30 min, washed with Tris-buffered saline-Tween20 (TBST), and probed with four kinds of primary antibodies at 4 °C overnight. β-actin was included as an internal loading control. After washing, the membrane was incubated with HRP-conjugated secondary antibody at RT for 1 h and washed again with TBST. The immunocomplex bands at PVDF membranes were detected using a ChemiDoc XRS (Bio-Rad, Hercules, CA, USA), and all Western blotting experiments were repeated three times. The intensity of density value (IDV) was analyzed using ImageJ software.

### Cell transwell migration assay

For evaluating the PI3K/Akt pathway involved in mediating the endothelial cell migration by activated OECs, the chemotactic motility of HUVECs was determined using Transwell migration chambers with 6.5 mm-diameter polycarbonate filters (8-µm pore size). As shown in Additional file 2: Fig. S2d, the lower chambers were filled with 600 µL of grouping media containing 5 μM MK 2206 or DMSO controls. HUVECs (3 × 10^4^ / well) were seeded in upper chambers in 100 µL serum-free DMEM. Cells were allowed to migrate for 8 h. Non-migrated cells were removed with cotton swabs, and migrated cells were fixed with ice-cold methanol and stained with 0.1% crystal violet. Using a Leica DM IL, cells were inspected under inverted light microscopy (20 ×). Images were captured by Leica LAS AF Lite software (Leica Microsystems, Germany) and quantified by Image J software.

### EdU incorporation assay

All operations were carried out according to the kit instructions. Briefly, HUVECs were seeded in the coverglass bottoms of 35 mm confocal dishes at a density of 1.5 × 10^4^ cells per dish along with EdU at a working concentration of 10 μM in 200 μL grouping media for 1 h. After being labeled by EdU, cells were fixed and permeabilized by fixative solution and permeabilization buffer at RT. The mixed reaction solution is prepared by mixing copper sulfate, iFour 488 Azide, and TBS. The reaction mixture was then added into each dish for 30 min at RT, followed by washing with PBS and staining with DAPI (1:1000) for 10 min at RT. After the staining, the cells were imaged under a fluorescent microscope, Leica DM IL, and quantified by Image J software. All assays were repeated three times.

### Statistical analysis

All data are presented as mean ± standard deviation (SD), and the statistical analyses were undertaken using GraphPad Prism7 software (La Jolla, USA). Statistical significance between groups was determined by analysis of variance (ANOVA). A Student’s t-test (normal distribution) was applied to compare the control and experimental groups. Statistical significance was defined as *P* < 0.05.

## Results

### Characterization and identification of OECs

**Fig. 1:**
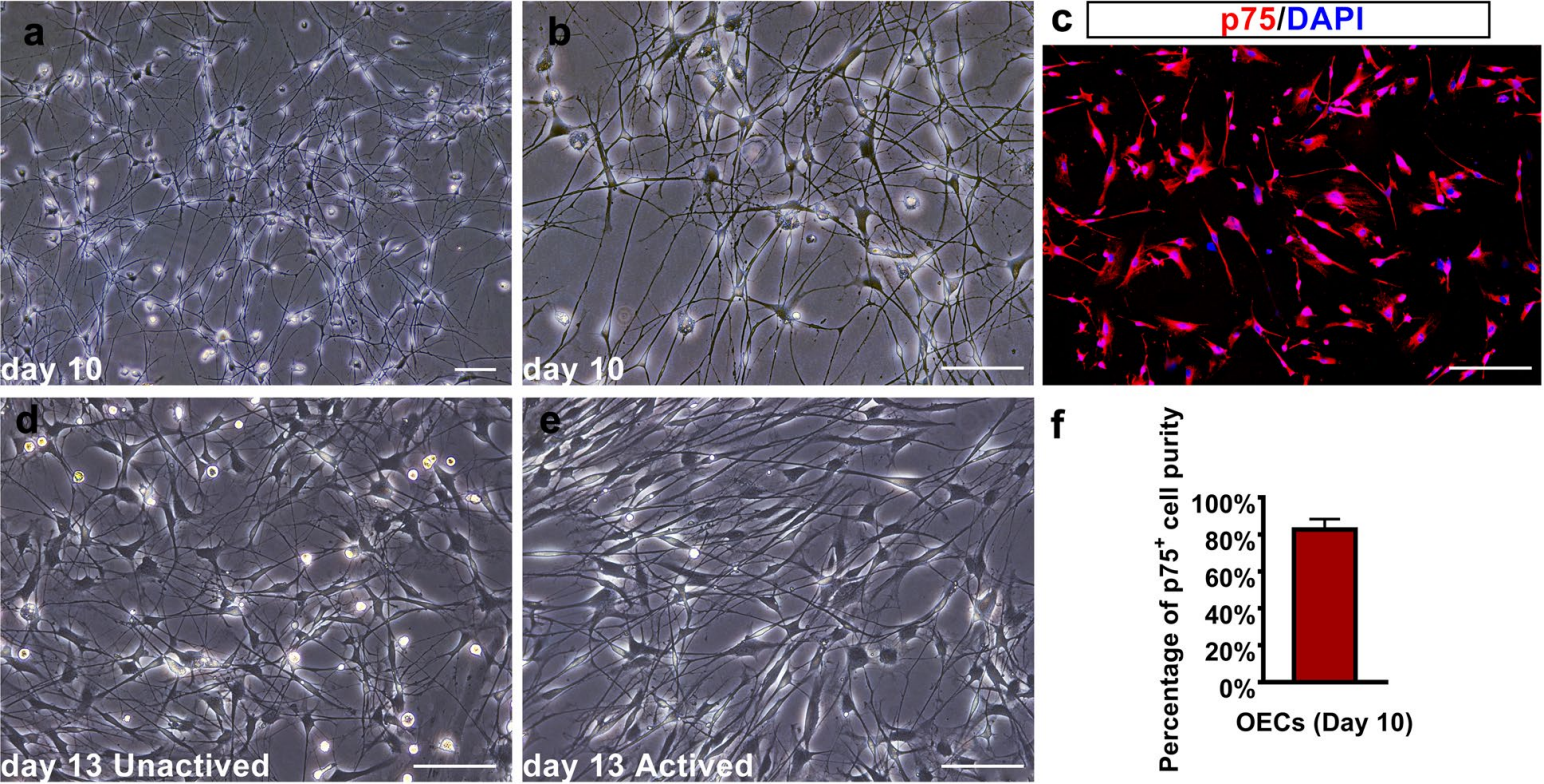
Characterization and identification of OECs. **a**, **b** Primary cultured OECs on day 10 under phase contrast microscopes. **c**, **f** Identification of immunocytochemistry for p75 and p75 positive cell purity of primary cultured OECs. **d**,** e** Primary cultured OECs on day 13 (three days after subculture) with or without activation under 20 × contrast microscopes. Scale bar: 100 μm

OECs were cultured and observed as described in the methods section. Ten days after primary culturing, the OECs were taken under inverted phase-contrast microcopy before subcultures. As demonstrated, the purified OECs have formed long, threadlike cellular processes connected to each other in a network (Fig. 1a). Observed under a 20 × magnification objective lens, the cell bodies displayed a bipolar, multipolar, or irregular shape (Fig. 1b). Before sub-culture, some OECs were digested and transferred to plastic coverslips for immunofluorescent imaging and identification. Immunocytochemical staining for p75 (a characteristic marker for OECs) demonstrated that over 85% of cells were OECs (Fig. 1c and f). After three days of sub-culturing and OEC activation, OECs were observed using a phase-contrast microscope. Both groups of cells were observed to have thicker processes than before sub-culturing. Upon observation of activated OECs, the cellular bodies of the OECs showed a more apparent bipolar morphology, and their processes extended in parallel directions compared to unactivated OECs. Subjectively, the whole cellular distribution of the activated OECs appeared isotropic (Fig. 1d and e).

### Effect of activated OECs-CM on biological behaviors of vascular endothelial cells

**Fig. 2:**
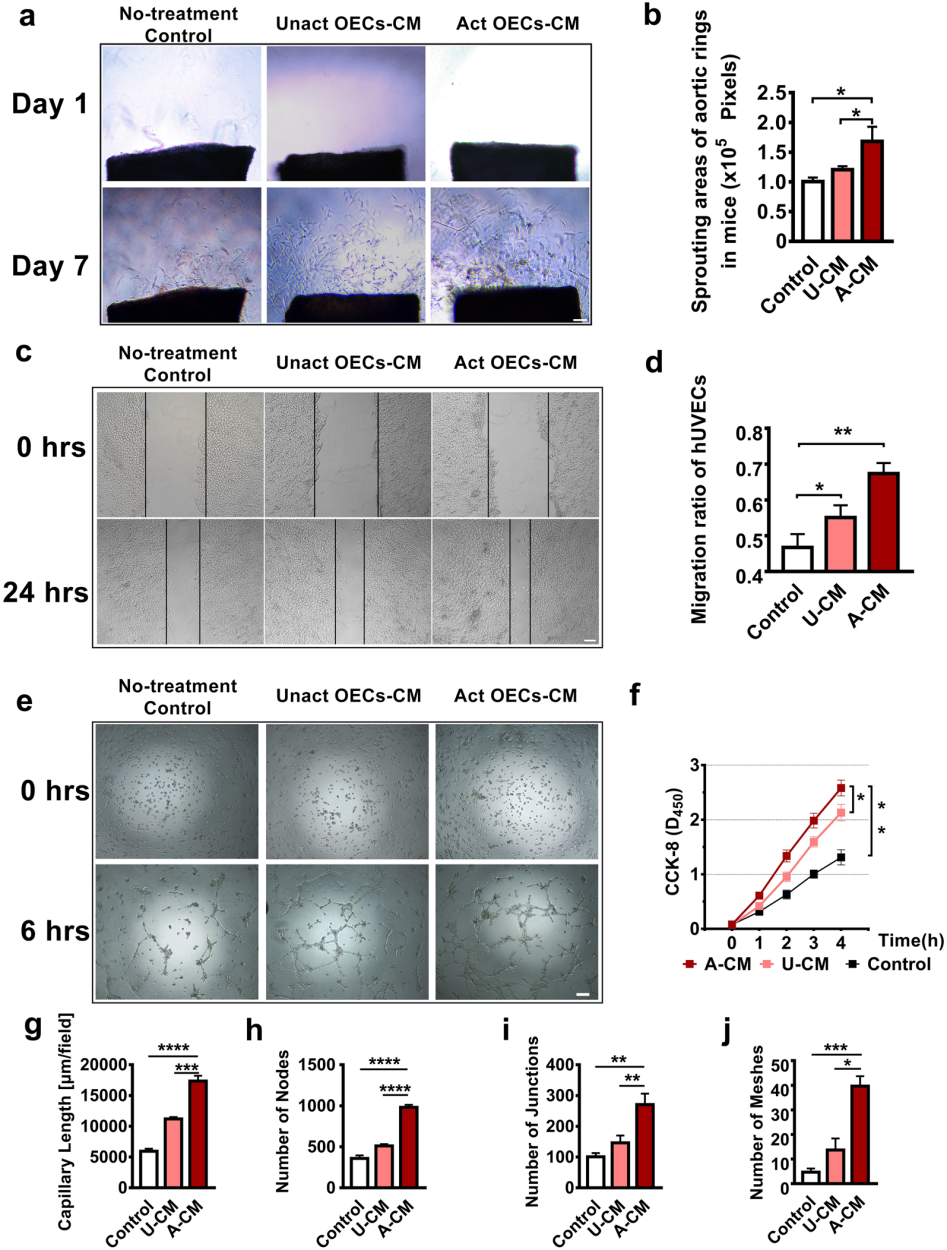
Effect of activated OEC-CM on biological behaviors of vascular endothelial cells. **a** In vitro representative photomicrograph of micro-vessels sprouted from young rat aortic rings embedded in Matrigel and incubated with OECs-CM or control media. Scale bar: 100 μm. **b** Quantitative analysis of sprout areas induced by incubation with each media group. **c** Phase-contrast micrographs of HUVECs at the initial time and 24 h following monolayer scratch wounding. Scale bar: 100 μm. **d** The migration of HUVECs within the scratch in the presence of different media groups. Healing index = (initial area—final area)/ initial area × 100%. **e** Representative images of HUVEC tube formation in vitro after culturing with OEC-CM or control media. Scale bar: 100 μm. **f** Effects of OECs-CM culture on cell proliferation by CCK-8 in HUVECs. **g**–**j** Quantitative evaluation of the number of capillary lengths, number of nodes, number of junctions (branch points), and number of meshes(loops) after treating HUVECs with OECs-CM. All data are reported as the mean ± SD of results from three independent experiments. *ACM* activated OECs-CM, *UCM* unactivated OECs-CM, n = 3; *P < 0.05, **P < 0.0*1*, ***P < 0.005, ****P < 0.001 vs the corresponding groups

The rat aortic ring assay was utilized to examine the neovascularization-promoting effect of OECs-CM in vitro. Using the variable-controlling approach, the CM collected from OECs induced the aortic ring to sprout, thereby emerging from the aorta and with a wide and dense network than observed with the non-conditioned media (*P* < 0.05; Fig. 2a and b). To confirm whether OEC CM induced endothelial migration, we cultured the HUVECs in control media and CM, using an in vitro scratch assay. After 24 h, HUVECs refilled the scratch areas, with the areas in three ACM groups being significantly narrow following the scratch assay. Although not as narrow as observed with the ACM group, there was a significant narrowing observed in the UCM group (Fig. 2c and d), which indicates that some substances promote the migration of vascular endothelial cells in OECs CM. From our CCK-8 assay results, the percentage of HUVEC proliferation in the ACM and UCM group was significantly increased compared to the control media group (ACM *vs.* control: *P* < 0.01; ACM *vs.* control: *P* < 0.05). Furthermore, the ACM group also had the most substantial proliferative effect (Fig. 2f). In contrast to the control media and UCM group, longer two-dimensional capillary walls and more tubular typical structures (number of nodes, junctions, and meshes) were formed on the matrix gel when HUVECs were cultured in ACM (Fig. 2e and g–j). This phenomenon of simulating vascular formation is an important experimental basis for carrying out our subsequent in vivo studies.

### Characterization and identification of RAECs

**Fig. 3:**
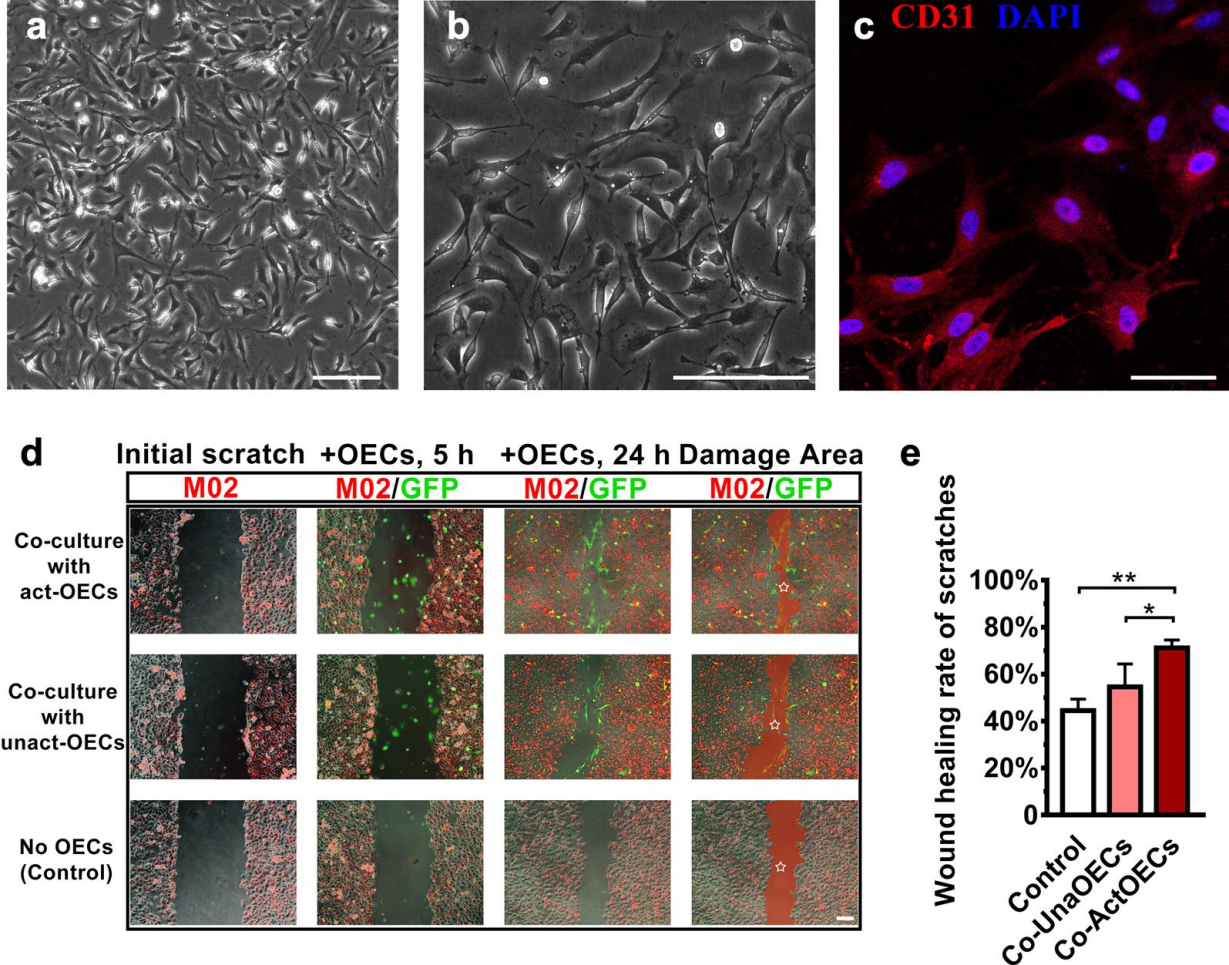
Effect of activated OECs on the healing of RAEC wound scratches by co-culture method in vitro. **a, b** Characterization of RAECs primary cultured on day 9 under the phase contrast microscopes. Scale bar: 100 μm. **c** Identification of immunofluorescence for CD31 of RAECs. Scale bar: 50 μm. **d** Simulated wound scratches of RAECs labeled with red fluorescent probe M02 (Initial stretches column) and co-cultured with OECs from GFP transgenic rats at different time points after OEC inoculation. The final scratch areas were covered with translucent red and marked by stars. Scale bar: 100 μm. **e** The wound healing rate for the three groups shown in **d**. Scale bar: 100 μm. The data are presented as the means ± SD; n = 3 per group. *P < 0.05, **P < 0.01 *vs* the corresponding groups

After inoculation for 72 h, HUVEC cells migrated to the aortic slices and grew adherent to the vascular fragments at the center. Cellular morphology was stable after 7 days, and the cells cultured on day 9 were observed under an inverted phase-contrast microscope. From this analysis, many cells were found to have migrated and adhered to plate (Additional file 3: Fig. S3h). The cells displayed a long spindle, or polygon, shape with bright halos at the edges, thereby displaying characteristics of flat epithelial cells (Fig. 3a and b). Immunocytochemical staining for anti-CD31 antibody (a characteristic marker for vascular endothelial cell) demonstrated that a large portion of the cells were endothelial cells (Fig. 3c).

### Effect of activated OECs on the healing of RAEC wound scratches in vitro

We prepared simulated wound scratches of RAECs in vitro labeled with a red fluorescent probe, M02. We then added OECs from GFP transgenic rats to establish a co-culture system (1st and 2nd columns of Fig. 3d). After 24 h of co-culture, images using fluorescence and bright field microscopes were collected and merged, and it was found that wound scratches in each group had displayed differences in healing characteristics (Fig. 3d). As for the comparison and analysis of the extent of reduction in the area of scratches, the RAECs incubated with activated OECs had the most significant degree of healing, followed by unactivated OECs in the co-cultured group (Fig. 3e, co-culture of activated OECs *vs.* control: *P* < 0.05; co-culture of activated OECs *vs.* co-culture of unactivated OECs: *P* < 0.01).

### Effect of OECs on angiogenesis after SCI in vivo

**Fig. 4:**
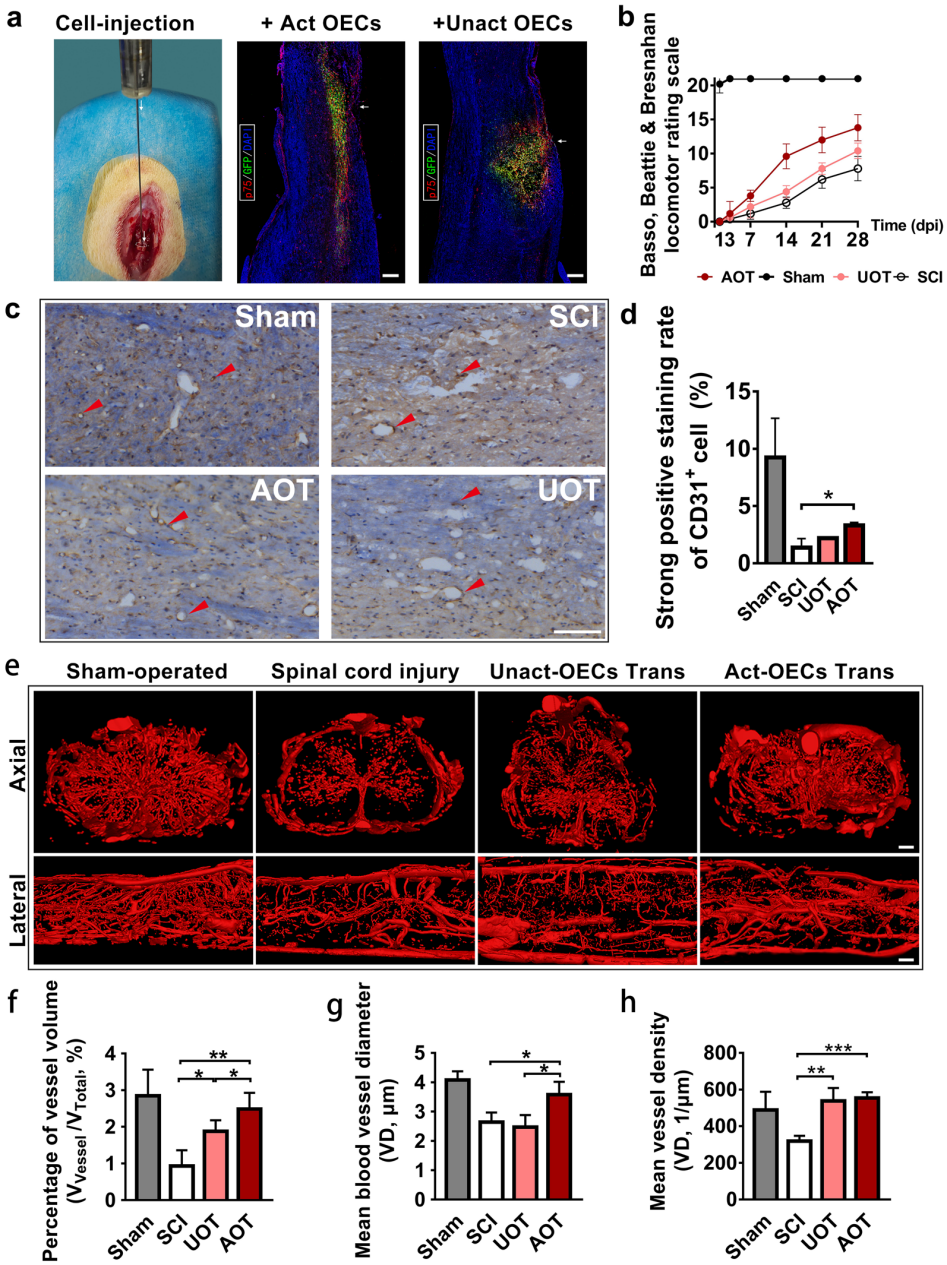
Effect of OECs on angiogenesis after SCI in vivo. **a** Injection of OECs from GFP transgenic rats into the spinal cord of rats, and immunofluorescent identification for p75 after one week. They display that all OECs were successfully transplanted to the injured areas and the activated OECs have a longer migration area. Scale bar: 100 μm. **b** BBB scores within the different observation periods in distinct treated rats after SCI. (n = 5 per group) **c** Representative images of immunochemical stained sections showing lumens surrounded by positive CD31 cells in the various groups (in vivo). Red arrows mark the typical CD31 positive cells. n = 3 per group, scale bars: 100 μm. **d** Positive staining rate of CD31^+^ cells. AOT: activated OECs transplantation group; UOT: unactivated OECs transplantation group. **e** Angiographic 3D reconstruction from Micro-CT photographs of spinal cords of rats one week after SCI, n = 3 per group. Scale bar: 200 μm. **f–h** The percentage of vessel volume (**f**), mean blood vessel diameter (**g**), and mean vessel density (**h**) calculated for the three groups shown in **d**. All data are reported as the mean ± SD of results. *P < 0.05, **P < 0.0*1*, ***P < 0.005 *vs* the corresponding groups

A subpopulation of the rats was sacrificed one week after SCI. Their spinal cord tissue was harvested and prepared into 10 μm slices and immunofluorescently labeled with anti-p75 antibodies. GFP and p75 positive cells displayed overlap, indicating high OEC-purity of the transplanted cells. From the sagittal view of the spinal cord, it is evident that the OECs were successfully transplanted to the injured area (Fig. 4a). Moreover, activated OECs were more widely distributed than unactivated OECs, and GFP-positive cells extended up and down longer (Fig. 4a and Additional file 4: Fig S4 e). The other subpopulations of rats (n = 5 per group) were observed for 28 days after SCI, and scoring resulting from the BBB scale was recorded. From comparisons of the locomotor performance of the control group, the animal model was successfully established, and the recovery of AOT was significantly faster than that of the UOT and SCI group (*P* < 0.05). On day 28, the BBB scores of the AOT, UOT, and SCI groups were 13.8 ± 1.92, 10.40 ± 1.14, and 7.80 ± 1.79. CD31-positive vascular endothelial cells of rats’ spinal cords were labeled by immunohistochemistry. To control for nonspecific staining, only the number of high-intensity CD31-positive stained cells were counted. As shown in Fig. 4 c and d, although the loss of vascular endothelial cells was severe in spinal cord injuries at week 1 (sham group *vs.* AOT/UOT/SCI group: *P* < 0.005), more CD31 positive cells were found in the AOT group than in the control group and SCI group yet (*P* < 0.05). To intuitively present the three-dimensional structure of spinal blood vessels, we performed contrast agent perfusion and angiography on each group of animals one week after SCI (Fig. 4e). In terms of the percentage of spinal vessel volume (Fig. 4f), there was no significant difference between the AOT group and the sham group one week after SCI (*P* > 0.05). These two groups were very similar in spinal vessel volume, while the vascular volume of the SCI and UOT groups was significantly smaller than that of the sham group (sham group *vs.* UOT group: *P* < 0.01; Sham group vs. SCI group: *P* < 0.05). Mean vessel diameter index and volume index had similar trends (Fig. 4g), in which the vessel lumen diameter of the AOT group was significantly larger than SCI and UOT groups (both *P* < 0.05). Also, it is worth noting that the average vessel lumen diameter of the UOT group treated with unactivated OECs-transplantation was 2.41 ± 0.41, which was slightly smaller than the vessel diameter of the SCI group with 2.64 ± 0.33. In terms of vascular density, both the AOT and UOT groups treated with OEC-transplantation were similar to the sham group (*P* > 0.05). The difference between the AOT and UOT groups was minor (*P* > 0.05), with more apparent blood vessel formation than the SCI group. Additionally, the vascular density was significantly higher than that of the SCI group (AOT group *vs.* SCI group: *P* < 0.001; UOT group *vs.* SCI group: *P* < 0.005).

### ACM and UCM contained angiogenesis-related growth factors

**Fig. 5:**
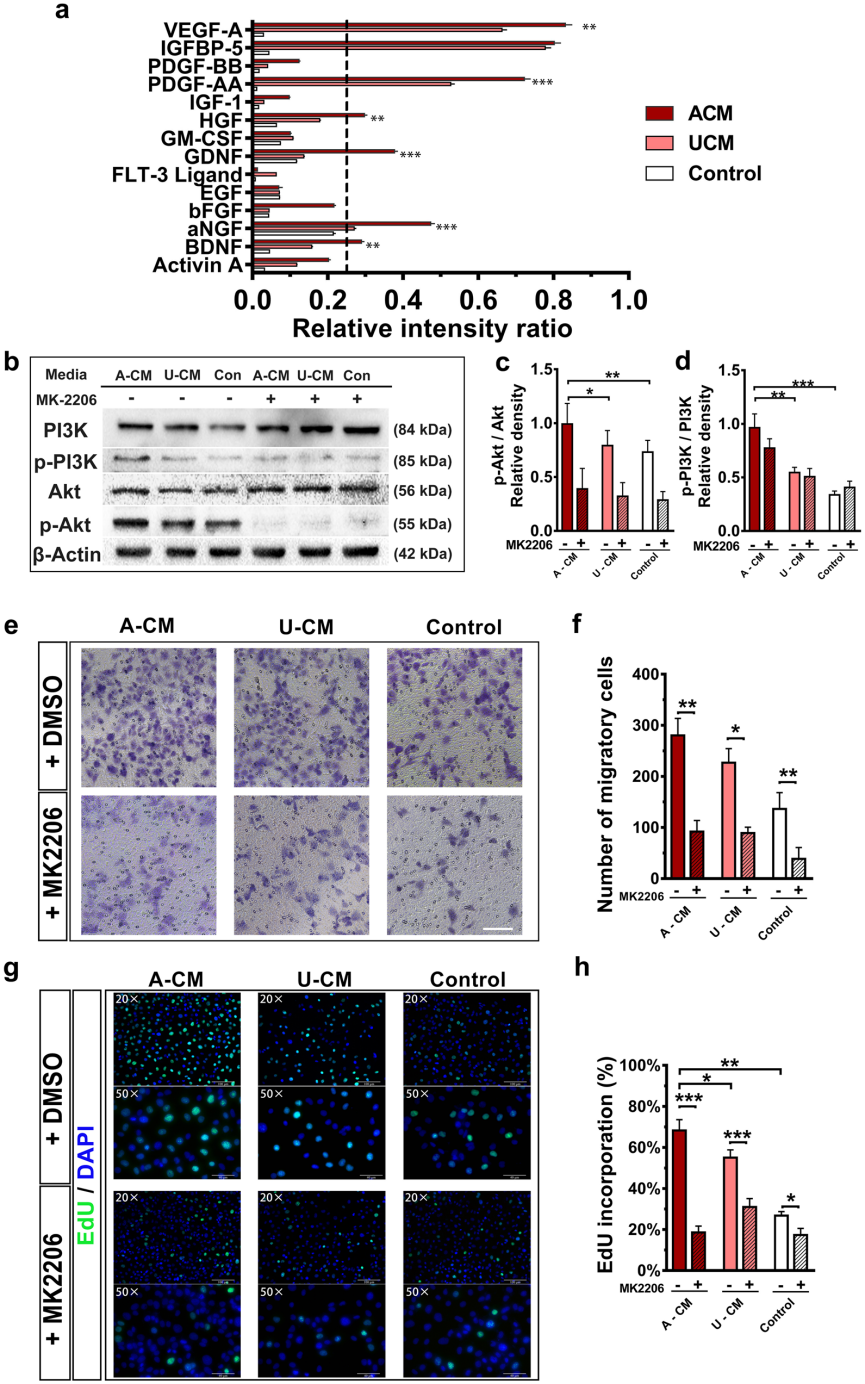
Possible molecular mechanism of the pro-angiogenic effects of Activated OECs. **a** Growth factor array for angiogenic factors. Each bar shows the relative intensity ratios of growth factors found in activated OECs-CM, unactivated OEC-CM, or control media, respectively. **b** Western blotting detected protein levels of PI3K, p-PI3K, Akt, and p-Akt in each group. **c** and **d** represent the relative density of phosphorylation level of Akt and PI3K, respectively. **e, f** OECs-CM and MK2206 effect on migration of HUVECs using Transwell migration chambers. Scale bar: 100 μm. **g, h** OECs-CM and MK2206 effect on proliferation of HUVECs using EdU incorporation assay. *ACM* activated OECs-CM, *UCM* unactivated OECs-CM. All values shown are the means ± SD (n = 3). *P < 0.05, **P < 0.01, ***P < 0.005 *vs* the corresponding groups

Activated OECs have been shown to promote angiogenesis in vivo and in vitro. To account for which angiogenic substances secreted by OECs promote this effect, OEC-CM was screened for angiogenic factors using a growth factor array to assess differences between ACM, UCM, and control media used in previous in vitro experiments. As shown in Additional file 6: Figure S6a–d and Fig. 5a, the growth factors with relative high enrichment levels (relative intensive ratio in each group > 0.25) and with significantly higher levels than the control group (*P* < 0.05) were found to include vascular endothelial growth factor A (VEGF-A), insulin-like growth factor-binding protein-5 (IGFBP-5), platelet-derived growth factor AA (PDGF-AA), hepatic growth factor (HGF), glial cell line-derived neurotrophic factor (GDNF), α-nerve growth factor (α-NGF) and brain-derived neurotrophic factor (BDNF). Among these identified growth factors, the relative enrichment level of VEGF-A PDGF-AA, HGF, GDNF, α-NGF, and BDNF in the ACM group was significantly higher than that in the UCM group. Of these growth factors, VEGF (for vascular endothelial cells) and PDGF (for vascular smooth muscle cells) are well-regarded as being beneficial in promoting angiogenesis [38, 39].

### Activated OECs promote angiogenesis through the PI3K/Akt signaling pathway

Previous studies have demonstrated that the activation of the PI3K/Akt pathway can promote the proliferation and migration of vascular endothelial cells. Furthermore, VEGF-A or PDGF -AA can mediate the activation of the PI3K/Akt pathway [40–42]. Therefore, combined with our screened growth factors, we hypothesized connections between activated OECs and the PI3K/Akt pathway. To address this, we added three Akt-inhibitory groups (MK2206 with Akt inhibitor) to evaluate differences in effects between the existing ACM, UCM, and control media groups. Detection of the phosphorylation levels of Akt and PI3K in the harvested lysates of HUVECs cultured in each respective media group was assessed by western blot analysis (Fig. 5b). We found that the phosphorylation levels in Akt and PI3K of HUVECs from the ACM group were significantly higher than those in the UCM and control groups (Fig. 5c and d). In addition, blotted bands in Fig. 5b and gray-value analysis of Fig. 5c also showed that MK2206 significantly inhibited the phosphorylation of Akt. Therefore, these results demonstrate that the phosphorylation level of the Akt/PI3K pathway was increased in activated OECs.

Next, we explored whether Akt/PI3K pathway activation could play a role in promoting angiogenesis. Cell motility was assessed with transwell migration assay by inoculating HUVECs in the upper chamber and grouped media in the lower chamber. We observed that the MK2206 supplemented media in the lower chambers induced less migration of HUVECs through the porous membranes, compared to their corresponding DMSO supplemented media control (ACM + DMSO group *vs.* ACM + MK2206 group: *P* < 0.01, UCM + DMSO group *vs.* UCM + MK2206 group: *P* < 0.05, Control + DMSO group *vs.* Control + MK2206 group: *P* < 0.01). Additionally, the EdU assay was used to analyze HUVEC proliferation following PI3K/Akt pathway inhibition. HUVECs cultured in MK2206 supplemented media resulted in a lower EdU incorporation rate than their corresponding DMSO supplemented media control (ACM + DMSO group vs. ACM + MK2206 group: *P* < 0.001, UCM + DMSO group *vs.* UCM + MK2206 group: *P* < 0.001, Control + DMSO group *vs.* Control + MK2206 group: *P* < 0.05). Similar to the results from the CCK-8 assay, it was found that HUVECs incubated in ACM had the most robust proliferative activity, as shown in Fig. 5g and h (ACM *vs.* UCM or Control group: *P* < 0.05). In contrast, the HUVECs cultured in MK2206 supplemented ACM were found to have significantly lower proliferation activity than those in MK2206 supplemented UCM group (ACM + MK2206 *vs.* UCM + MK2206: *P* < 0.05). In summary, our results suggest that activated OECs can secrete angiogenesis-related growth factors and promote angiogenesis through the activation of the PI3K/Akt pathway.

## Discussion

The limited repairability of neurons and the secondary deterioration of the microenvironment are some of the main causes of functional recovery difficulties following a SCI [43]. The blood supply is closely related to changes in the microenvironment of the injured area. Primary mechanical trauma from SCI leads to the rupturing and shearing of the microvascular and the BSCB in the surrounding tissue, which can subsequently cause an imbalance of hemorrhage and ischemia, thereby leading to pathological changes such as inflammatory infiltration and edema of the nerve tissue environment [17]. Intact vascular structures and a rich blood supply can promote oxygen/nutrient delivery and the removal of metabolic waste. Thus, the vasculature of the spinal cord plays a crucial role in SCI and repair processes. Otherwise, tissue repair can be hindered and deteriorate without the proper supply of nutrients from the blood [44, 45]. Furthermore, a massive loss of endothelial cells can occur acutely (during the first 24-h) due to the impact-generated mechanical forces resulting from necrosis and ischemia-induced apoptosis that may follow the acute injury [46]. As the most important cells in the vasculature and effective promoters of angiogenesis, vascular endothelial cells should be considered in the early stage of cell therapy intervention for patients with SCI, with the potential of reducing secondary damage and promoting a microenvironment conducive to nerve repair. To begin exploring this strategy, we investigated OECs as an essential candidate for SCI repair to support this method for early-stage-angiogenesis in the spinal cord following an injury.

**Fig. 6:**
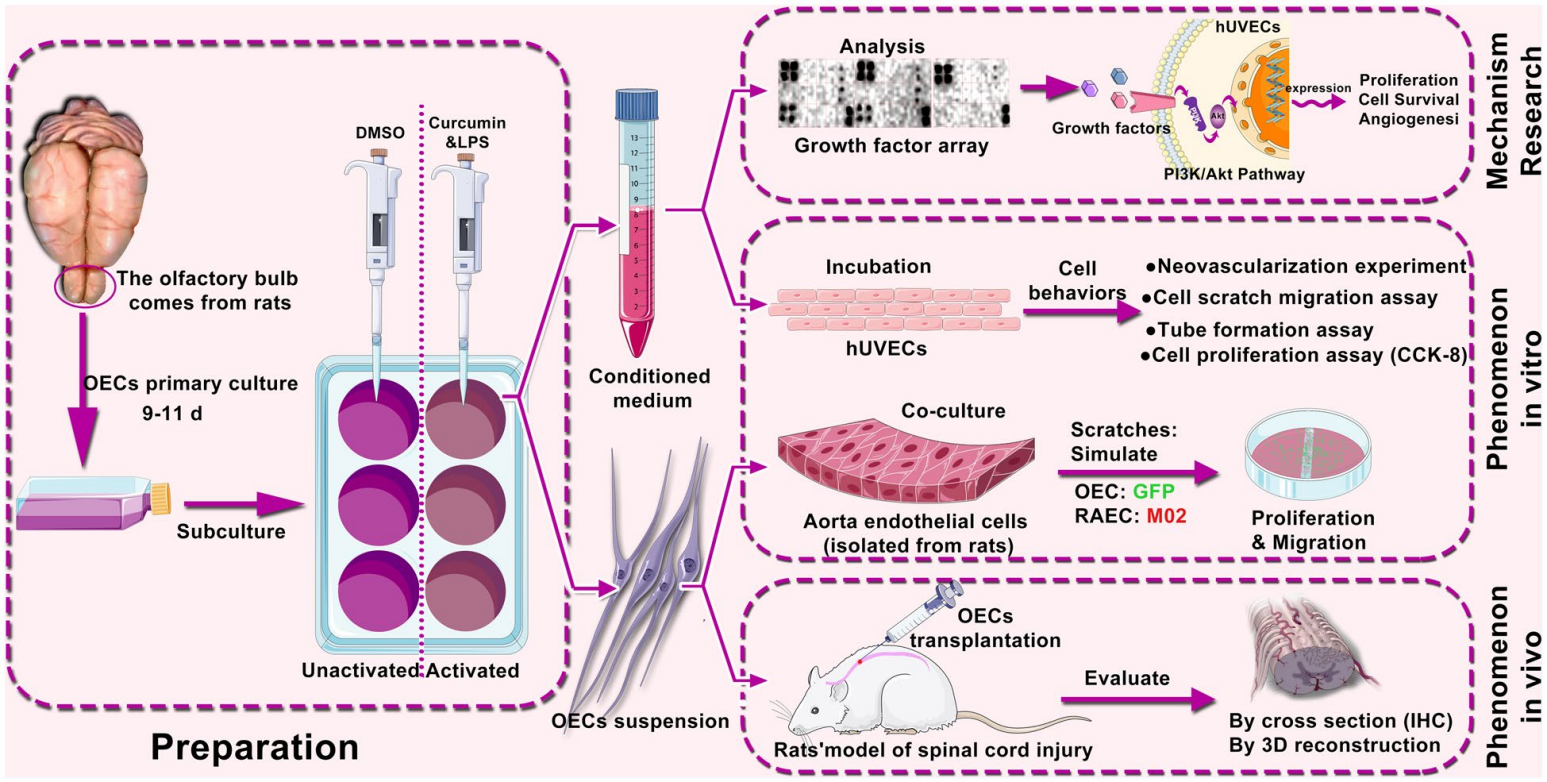
Schematic diagram showing the overall steps of our experiment and the promoting effects of activated OECs on angiogenesis after SCI through the PI3K/Akt pathway. The effects were observed in vitro and in vivo using CM and cell suspension as interveners separately. In addition, the mechanism behind these effects is revealed to a certain extent under the inspiration from some growth factors screened out by growth factor arrays

In the present study, we used previously published activation processing methods to reinforce the function of OECs in this strategy [15]. As shown in Fig. 6, we observed the indirect and direct effects of OECs on the biological behaviors of vascular endothelial cells using OEC-CM and OEC suspensions, respectively. We found that OEC-CM can induce neovascular sprouting from the broken ends of blood vessels, promote the proliferation and migration of endothelial cells, and form the two-dimensional structure of capillary-like lumen.

To better assess this complex investigation in vivo, we designed a co-culture-scratch-wound system of OECs and RAECs, including the cultured media environment without mitomycin, to assess proliferation and migration. We found that OECs promoted endothelial cell proliferation and migration in a direct contact environment using these co-culture systems. It is worth emphasizing that both the activated OECs and their CM showed significant biological effects in all groups. Notably, these findings complement our previous investigations that identified this biological potential of OECs activated by LPS & CCM [14].

According to our rat aortic ring assay results and previous reports [30, 31], we designated the animals to be sacrificed one week after injury, including the isolation of spinal cords. After validating our SCI injury and cell transplantation model, we used immunohistochemistry and micro-CT angiography to analyze the details of spinal angiogenesis, specifically looking at the number of endothelial cells and effective vessels in two and three dimensions, respectively. Our immunohistochemical investigations identified a significant proliferation in the AOT groups. However, many endothelial cells were still lost in the spinal cord after compressional injury, in which we found fewer CD31^+^ endothelial cells in the experimental group than in the sham group. Surprisingly, micro-CT angiography results showed that the AOT, UOT, and sham groups were similar in vessel volume and number. The loss of endothelial cells after tissue injury and the near-normal vascular structure may seem paradoxical, but it is consistent with the early manifestations of angiogenesis. Our results may reveal that the processes of angiogenesis promoted by OECs were more dependent on endothelial cell migration than their proliferation in the injured area. The migration of native cells from the wound boundary is critical in the wound healing process. Therefore, endothelial cell migration is an essential part of angiogenesis at the background of tissue lesion [47]. To some extent, this can explain why there are differences in the repair process of cells (CD31^+^ cell rate) and tissues (angiography) using our established in vivo model. Furthermore, the amplitude difference between proliferation and migration is worth further investigation as this can further support the differences in repair processes.

Previous studies have found that different subtypes of endothelial cells play different roles in new blood vessel formation, with some more responsible for migration guidance and others for proliferation. For instance, endothelial “tip cells” at the forefront of a sprouting vessel navigate by extending filopodia to act as the formation-spearhead, which is mainly responsible for migration and guide. “Stalk cells” trail behind these extensions and elongate the branch, which can then proliferate under the guidance of the tip [48]. Collectively, these findings suggest that the tips of the sprouts are composed of highly migratory cells. Notably, tip cells are suggested to mediate sprouting initiation at the broken ends of blood vessels with migration potential rather than proliferative potential. The stalk cells that follow the tip cells build the body of the growing sprout and, after spatial rearrangement, will form a lumen where blood can flow. Hence, the endothelial cells’ migration chronologically precedes proliferation, and this process is more pronounced in vivo. Therefore, the determination of proliferation (e.g., CCK-8 array) and migration ability (e.g., wound healing or aortic ring assay) under the premise of endothelial subtype differentiation and time-continuous observations can help define the pattern of angiogenesis in the SCI, and this can provide a theoretical basis for the sequential clinical treatment of SCI to some extent. Therefore, the exact structural pattern changes of angiogenesis after SCI should be explored in future studies to elucidate the progressive relationship between migration and proliferation.

Investigations from this study convincingly demonstrate that OECs, especially activated OECs, can indirectly and/or directly promote endothelial cell proliferation and migration and induce neovascularization. Importantly, endothelial cell migration is an integral part of the earlier steps in the angiogenic cascade, in which a group of cells coordinates their movements toward a chemotactic gradient and establishes a clear hierarchy of both leader and follower cells [49]. This pattern was named Connective Cell Migration, and the two most essential elements of this process are chemokines and leadership cells (tip cells) [50]. Hence, an important remaining question revolves around identifying which chemokines and/or growth factors mediated by activated OECs promote spinal angiogenesis.

To identify key chemokines and/or growth factors, we used the binding reaction of membranes (preloaded growth factors binding target) and OEC-CM and identified a high expression of VEGF-A and PDGF-AA in the activated group. Angiogenesis, the process of new blood vessel formation, is critical during development and subsequent physiologic homeostasis. The current academic consensus is that VEGF-A is one of the most significant growth factors for vascular development and angiogenesis stimulation [51, 52]. Likewise, PDGF-AA is a potent mitogen and chemoattractant for smooth muscle cells and fibroblasts in culture, which can induce the directed migration and proliferation of arterial smooth muscle cells and fibroblasts [53, 54]. Among the participant cells, endothelial cells are necessary to form new vessels and display a remarkable capability to switch rapidly from a quiescent state to a highly migratory and proliferative state during vessel sprouting [55]. Thus, our investigations into these mechanisms used endothelial cell lines as the object of intervention and observation.

The activation of the PI3K/Akt pathway has been shown to stimulate several essential cellular responses intrinsic to angiogenesis, such as survival, migration, and tube formation. Furthermore, one of the essential pathways to initiate these responses is the binding of VEGF-A to VEGFR-2, which activates the receptor’s kinase activity and engages PI3K and its downstream effector Akt [39, 56, 57]. Prompted by our array screening results, we wanted to confirm the involvement of this pathway during angiogenesis following injury to the spinal cord. Our investigations found that this pathway is involved as measured by kinase phosphorylation levels (Western blotting) and by indirect comparative observation via pathway inhibition (Transwell migration and Edu incorporation assay). From these studies, our results confirm that the activation of the PI3K/Akt pathway is an important mechanism contributing to OEC promotion of angiogenesis.

Interestingly, the classical PI3K/Akt pathway is well known to be involved in multiple processes and responses. In regards to neurons., several studies have found that the PI3K/Akt signal pathway critically mediates neuroprotection, axonal regeneration, and neurogenesis [58, 59]. Concerning OECs, the activation of PI3K/Akt signal pathway can protect and reinforce the OECs in the form of feedback mechanisms, such as promoting migration, survival, and proliferation [60, 61]. As for the immune microenvironment, this pathway can prevent neural injury by modulating microglia/macrophage polarization and antioxidant effects [62, 63]. Lastly, from the perspective of stem cell transplantation, the activation of the PI3K/Akt pathway by exogenous intervention can promote differentiation of endogenous neural stem cells and even exogenous stem cells with SCI-treatment, as observed with spermatogonial stem cells [64–66]. Taken together, the mechanisms of OECs promoting the repair of nervous system injury are indeed multifaceted, and our present study partially elucidates these complex mechanisms. Collectively, these findings demonstrate the comprehensive advantages of cell therapy and our exploration of activation intervention and other potentials for OECs as a theoretical supplement to cell therapy. In alignment with our research strategy, the core idea of sequential cell transplantation therapy is to improve the microenvironment with OECs before repairing and replenishing neurons. Sufficient angiogenesis and suppressed inflammatory responses induced by transplanted OECs can facilitate the microenvironment at the injured site and serve to pioneer future investigations into the use of OECs for SCI. With these investigations and strategies in mind, we speculate that it is conducive to differentiate exogenous stem cells into neurons, thereby leading to significant improvements in the efficiency of nerve repair.

## Conclusion

Our study observed changes of vascular endothelial cells and tissues after OEC intervention in vivo and in vitro. These mechanisms were found to involve indirect effects using conditioned media from OECs and direct effects resulting from co-culture and cell transplantation studies, in addition to findings from protein array screenings and mechanism-related experiments leading to the identification of the PI3K/Akt pathway being involved in these processes. Our study further confirms the pro-angiogenic potential of OECs in treating SCI and lays the foundation for our sequential therapeutic concept of microenvironment amelioration-stem cell transplantation.

## Supplementary Information

**Additional file 1: Fig. S1**. The process of olfactory bulb isolation. a Cut the scalp of a rat along the midline from neck to nose. b Remove exposed pieces of skull. c Olfactory bulb was placed in PBS.

**Additional file 2: Fig. S2**. Supplementary images of endothelial cell behavior tests. a Representative images of HUVECs under inverted phase contrast microscopes. b Method diagram of rat aortic ring assay. c Method diagram of HUVECs tube formation assay. d Method diagram of cell transwell migration assay.

**Additional file 3: Fig. S3**. The process of aorta slides isolation and primary culture of RAECs. a-g The process of aorta slides isolation detailed in ‘Materials and Methods’. h Characterization of RAECs primary cultured on day 9 under phase contrast microscopes.

**Additional file 4: Fig. S4**. Surgical procedure and morphological results of a rat model of compressional SCI. a-c The process of the surgical procedure detailed in ‘Materials and Methods’. d Hematoxylin and eosin staining image of a longitudinal section of the spinal cord at one week after SCI. e Longitudinal range of OEC’s survival area in frozen sections of the spinal cord in rats (n=5).

**Additional file 5: Fig. S5**. Preparation for spinal cord angiography. a Microfil® MV-122 (Yellow) silicone rubber contrast angiography agents were used in this study. b Perfusion process of angiographic agent c a sign of successful perfusion: yellow-stained sclera of the rat. d-g Spinal cord segments isolated after perfusion belonged to activated OECs transplantation, unactivated OECs transplantation, SCI, and sham-operated group, respectively.

**Additional file 6: Fig. S6.** a-c Representative image of growth factor assays of each group. a activated OECs-CM group; b unactivated OECs-CM group; c control media group. d indicating a map of the growth factor assay. e, f HUVECs were cultured in 6-well plates with or without MK2206 separately in each media group, and their protein samples were extracted.

## Data Availability

The datasets supporting the conclusions of this article are available in the PubMed repository (https://pubmed.ncbi.nlm.nih.gov).
