# Supplementary figures and images for: The promoting effects of activated olfactory ensheathing cells on angiogenesis after spinal cord injury through the PI3K/Akt pathway
Source: Cell Biosci. 2022 Mar 4;12:23. doi: 10.1186/s13578-022-00765-y (PMC8895872; doi:10.1186/s13578-022-00765-y)

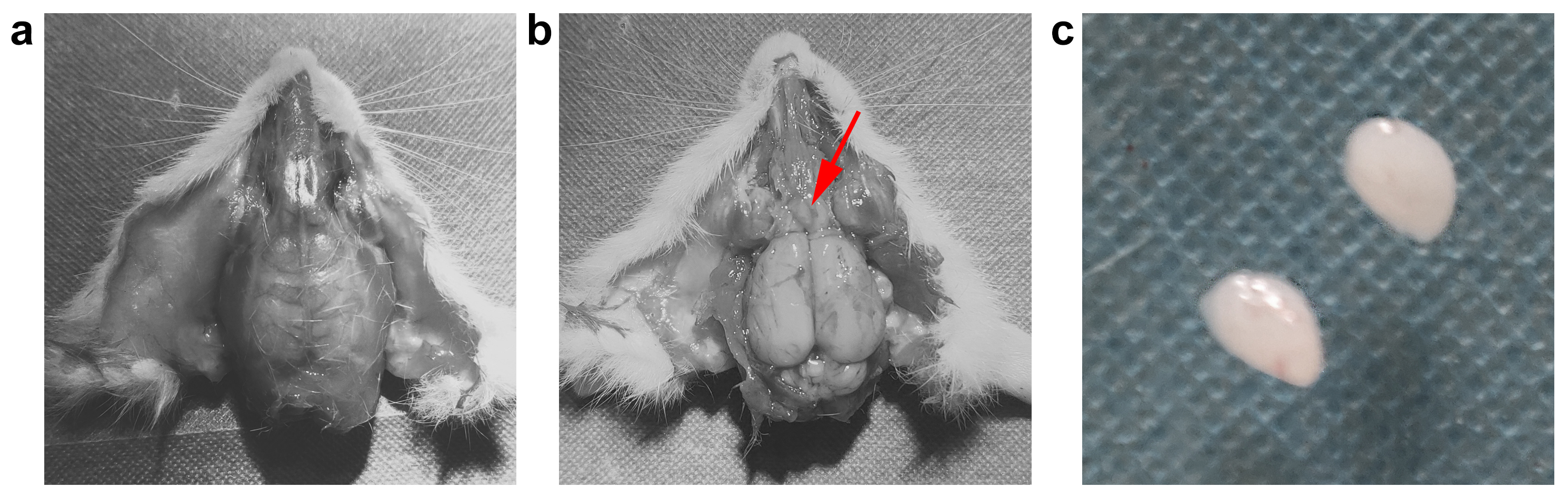

Supplement: Supplementary file 1 — Additional file 1: Fig. S1. The process of olfactory bulb isolation. a Cut the scalp of a rat along the midline from neck to nose. b Remove exposed pieces of skull. c Olfactory bulb was placed in PBS. [file 13578_2022_765_MOESM1_ESM.jpg]

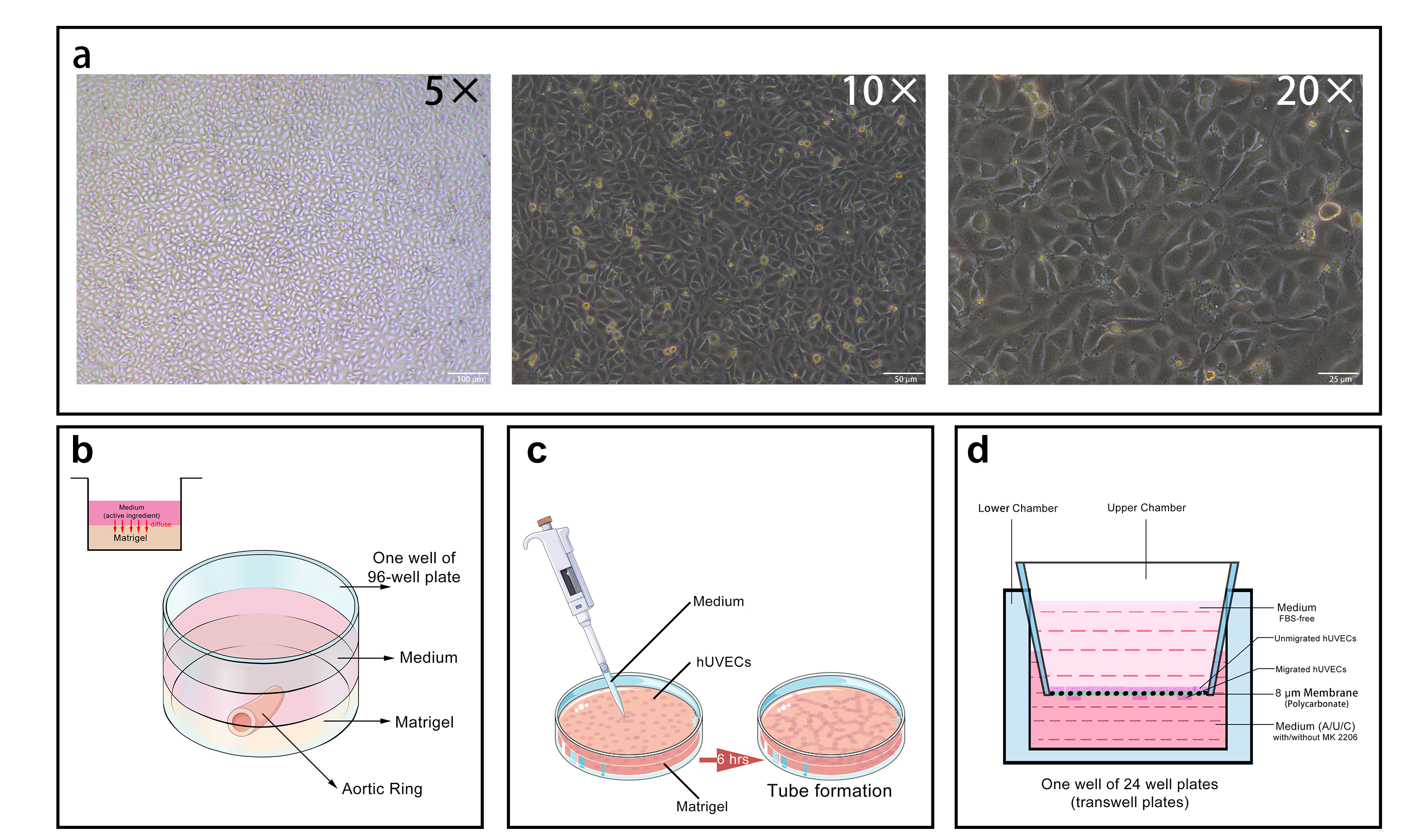

Supplement: Supplementary file 2 — Additional file 2: Fig. S2. Supplementary images of endothelial cell behavior tests. a Representative images of HUVECs under inverted phase contrast microscopes. b Method diagram of rat aortic ring assay. c Method diagram of HUVECs tube formation assay. d Method diagram of cell transwell migration assay. [file 13578_2022_765_MOESM2_ESM.jpg]

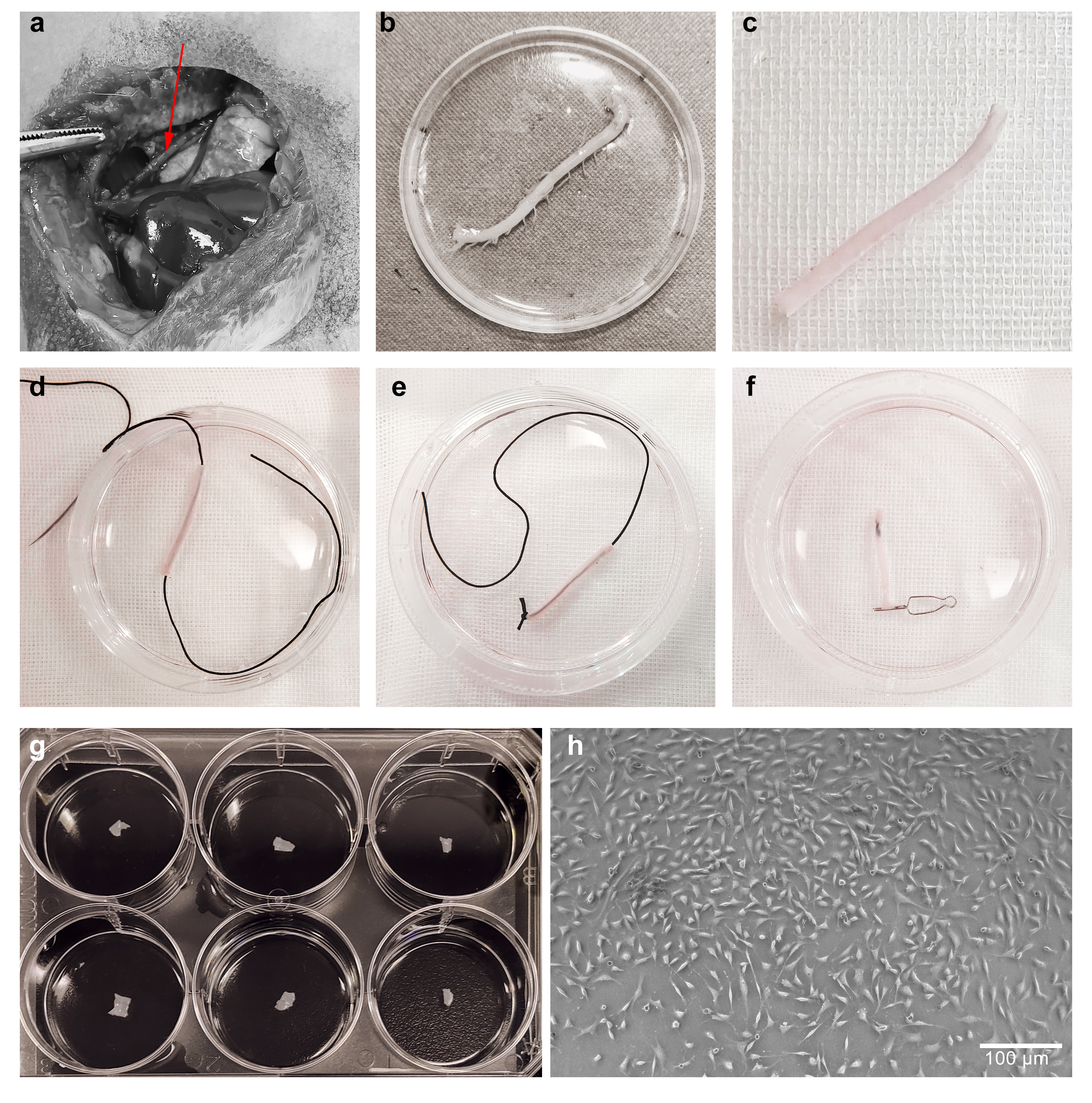

Supplement: Supplementary file 3 — Additional file 3: Fig. S3. The process of aorta slides isolation and primary culture of RAECs. a-g The process of aorta slides isolation detailed in ‘Materials and Methods’. h Characterization of RAECs primary cultured on day 9 under phase contrast microscopes. [file 13578_2022_765_MOESM3_ESM.jpg]

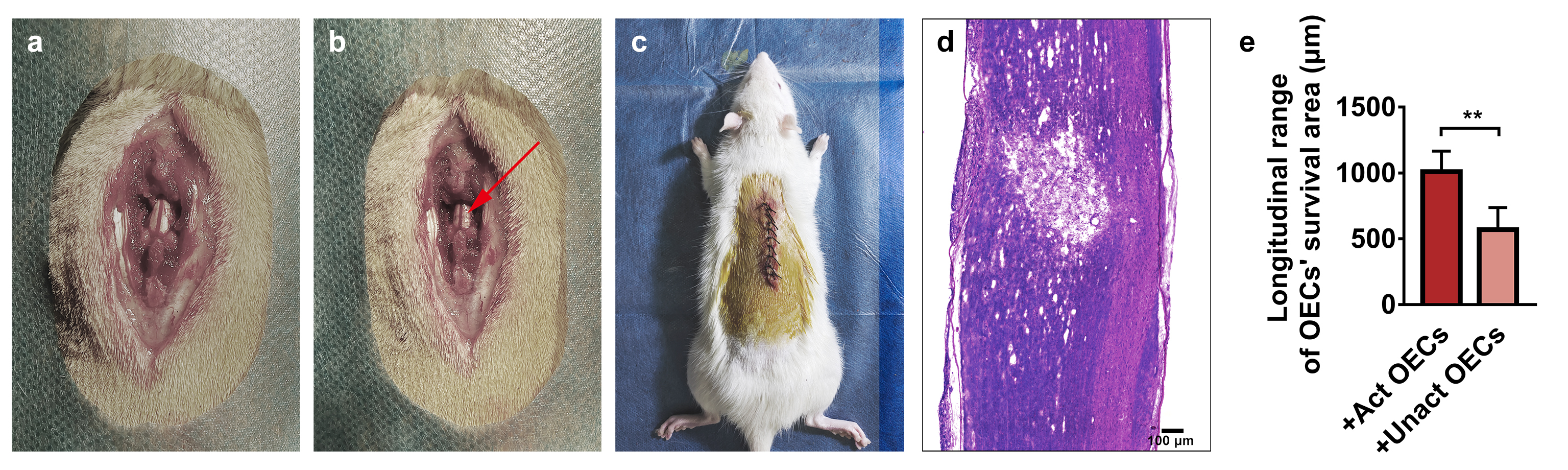

Supplement: Supplementary file 4 — Additional file 4: Fig. S4. Surgical procedure and morphological results of a rat model of compressional SCI. a-c The process of the surgical procedure detailed in ‘Materials and Methods’. d Hematoxylin and eosin staining image of a longitudinal section of the spinal cord at one week after SCI. e Longitudinal range of OEC’s survival area in frozen sections of the spinal cord in rats (n=5). [file 13578_2022_765_MOESM4_ESM.jpg]

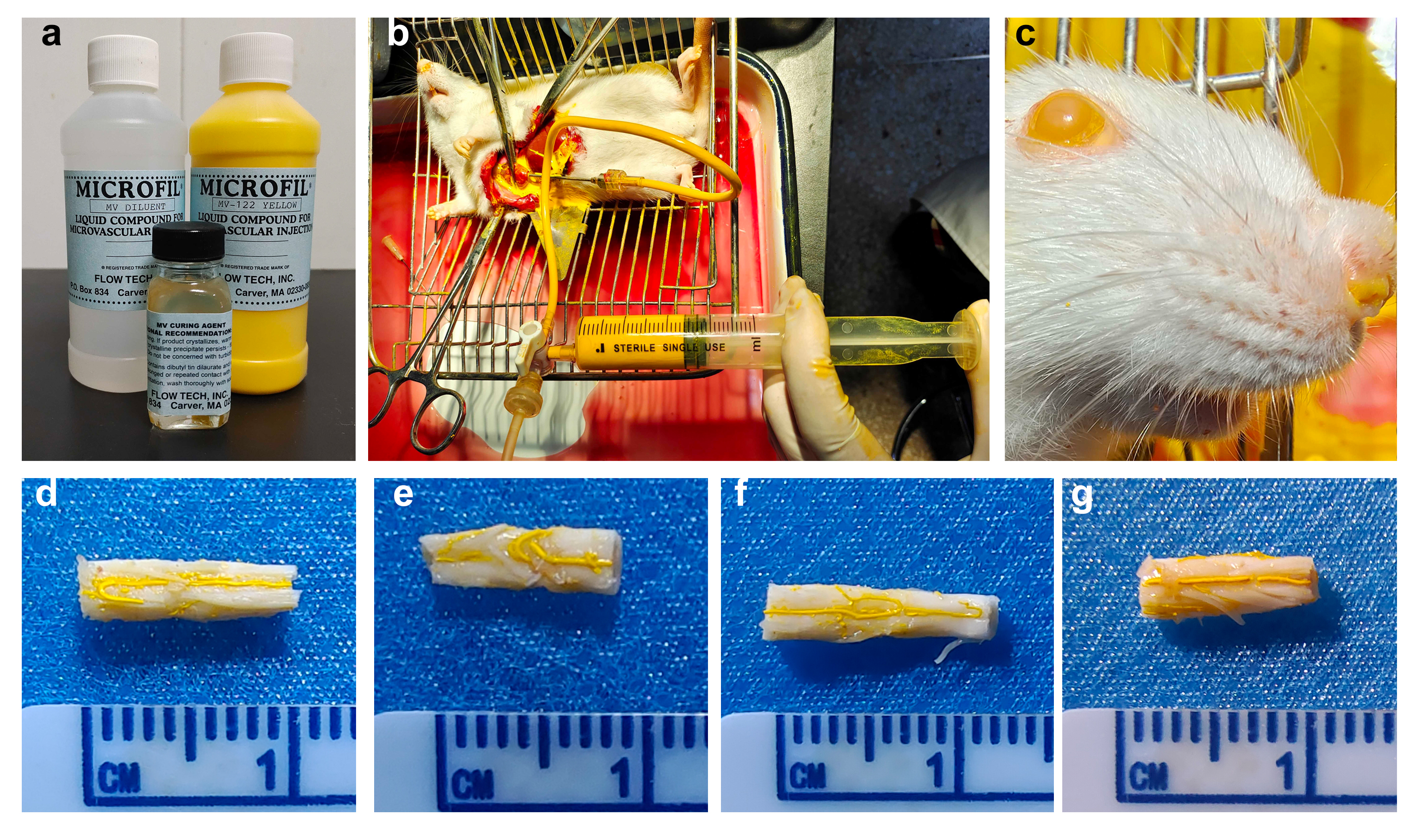

Supplement: Supplementary file 5 — Additional file 5: Fig. S5. Preparation for spinal cord angiography. a Microfil® MV-122 (Yellow) silicone rubber contrast angiography agents were used in this study. b Perfusion process of angiographic agent c a sign of successful perfusion: yellow-stained sclera of the rat. d-g Spinal cord segments isolated after perfusion belonged to activated OECs transplantation, unactivated OECs transplantation, SCI, and sham-operated group, respectively. [file 13578_2022_765_MOESM5_ESM.jpg]

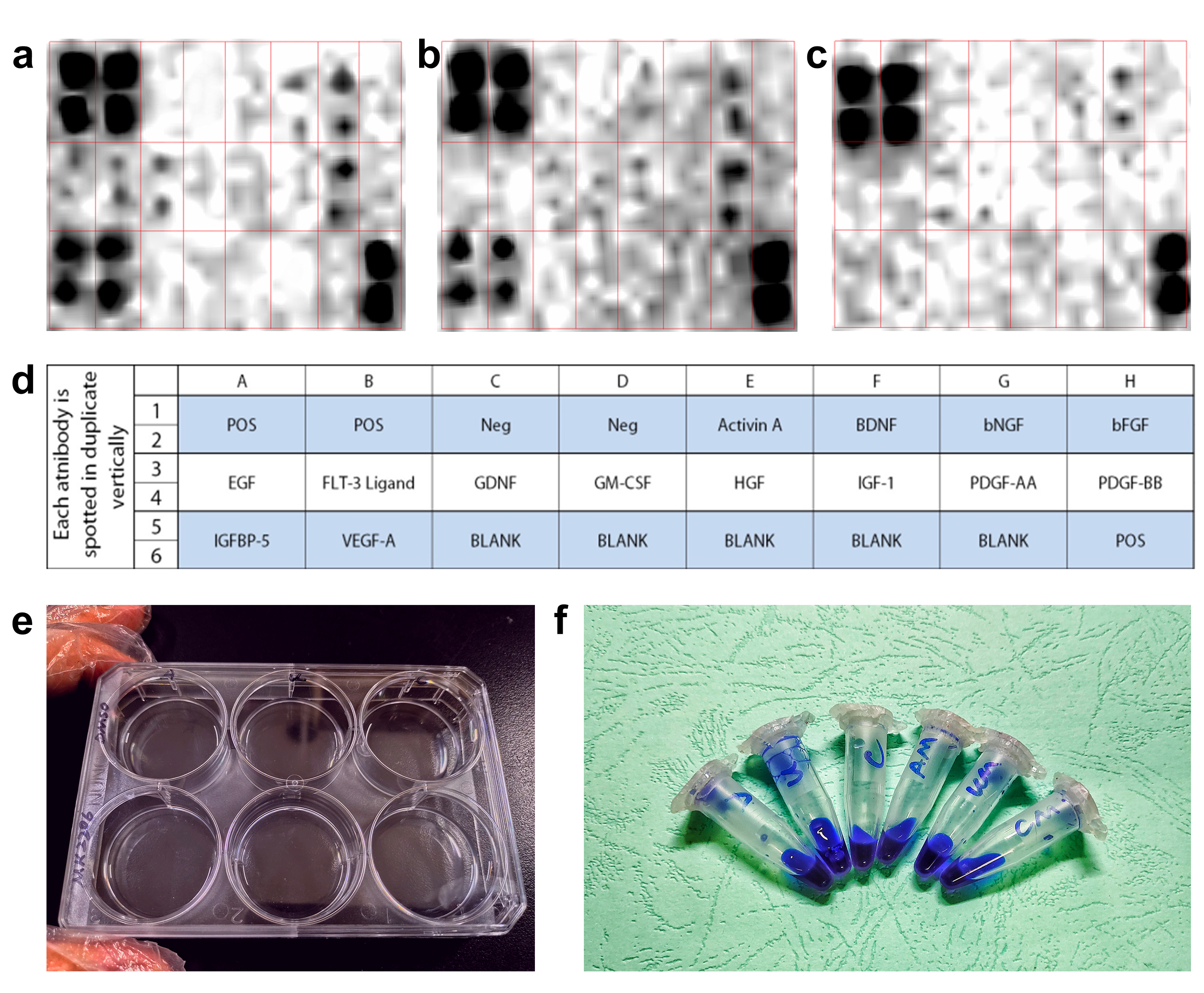

Supplement: Supplementary file 6 — Additional file 6: Fig. S6. a-c Representative image of growth factor assays of each group. a activated OECs-CM group; b unactivated OECs-CM group; c control media group. d indicating a map of the growth factor assay. e, f HUVECs were cultured in 6-well plates with or without MK2206 separately in each media group, and their protein samples were extracted. [file 13578_2022_765_MOESM6_ESM.jpg]
